# Assessment of the Adaptive Force of Elbow Extensors in Healthy Subjects Quantified by a Novel Pneumatically Driven Measurement System with Considerations of Its Quality Criteria

**DOI:** 10.3390/diagnostics11060923

**Published:** 2021-05-21

**Authors:** Silas Dech, Frank N. Bittmann, Laura V. Schaefer

**Affiliations:** Regulative Physiology and Prevention, Department of Sports and Health Sciences, University of Potsdam, 14476 Potsdam, Germany; bittmann@uni-potsdam.de (F.N.B.); lschaefe@uni-potsdam.de (L.V.S.)

**Keywords:** adaptive force, neuromuscular functionality, sensorimotor control, isometric muscle action, eccentric muscle action, maximal voluntary contraction, adaptive holding capacity, reliability, validity

## Abstract

Adaptive Force (AF) reflects the capability of the neuromuscular system to adapt adequately to external forces with the intention of maintaining a position or motion. One specific approach to assessing AF is to measure force and limb position during a pneumatically applied increasing external force. Through this method, the highest (AF_max_), the maximal isometric (AFiso_max_) and the maximal eccentric Adaptive Force (AFecc_max_) can be determined. The main question of the study was whether the AFiso_max_ is a specific and independent parameter of muscle function compared to other maximal forces. In 13 healthy subjects (9 male and 4 female), the maximal voluntary isometric contraction (pre- and post-MVIC), the three AF parameters and the MVIC with a prior concentric contraction (MVICpri-con) of the elbow extensors were measured 4 times on two days. Arithmetic mean (M) and maximal (Max) torques of all force types were analyzed. Regarding the reliability of the AF parameters between days, the mean changes were 0.31–1.98 Nm (0.61%–5.47%, *p* = 0.175–0.552), the standard errors of measurements (SEM) were 1.29–5.68 Nm (2.53%–15.70%) and the ICCs(3,1) = 0.896–0.996. M and Max of AFiso_max_, AF_max_ and pre-MVIC correlated highly (r = 0.85–0.98). The M and Max of AFiso_max_ were significantly lower (6.12–14.93 Nm; *p* ≤ 0.001–0.009) and more variable between trials (coefficient of variation (CVs) ≥ 21.95%) compared to those of pre-MVIC and AF_max_ (CVs ≤ 5.4%). The results suggest the novel measuring procedure is suitable to reliably quantify the AF, whereby the presented measurement errors should be taken into consideration. The AFiso_max_ seems to reflect its own strength capacity and should be detected separately. It is suggested its normalization to the MVIC or AF_max_ could serve as an indicator of a neuromuscular function.

## 1. Introduction

Forces which are generated by human muscles are generally related to the strength of a person, e.g., maximal strength, strength endurance or power [1]. In addition to such common measures, Adaptive Force (AF) was introduced recently [2,3,4,5]. AF not only requires muscle strength but also sensorimotor control. It reflects the neuromuscular functionality of adapting adequately to external forces with the intention of maintaining a desired position or movement. Thereby, the external force can be constant or vary in size. The adaptation to constant loads can be measured by holding a defined weight or resisting an applied constant force isometrically in a specific joint angle. This is termed “position task” [6,7], or “eccentrically loaded isometric contraction” [8], with an underlying “holding isometric muscle action” (HIMA) [9]. 

However, in daily activities and sports, persons have to deal mainly with external forces which vary in size. The specific task of adapting to varying external forces has been rarely considered in sports or movement science yet [2,3,4,5]. Such adaptation implies an evident relevance of avoiding an inappropriate lengthening of muscles, accompanied by a destabilization of joints despite an external force impact. This plays an important role, especially during an increasing force when a position, object or the moving body should be held or decelerated (e.g., during landing phases while running, descending stairs or side cutting maneuvers; resisting a tackle). An inadequate adaptation to increasing forces might result in impaired joint stability. As a possible consequence, injuries or damages of muscles, tendons and joints might occur [2,4,10,11]. For this reason, assessing AF could be a novel approach to understand the mechanisms behind it and to derive preventive strategies.

To measure adaptation to varying or increasing forces, no common measurement procedure is currently available. Although isokinetic devices can apply varying forces, these serve generally to keep the desired movement velocity constant. Thereby, the isokinetic device adapts to the generated force of the person. However, in capturing the AF to varying or increasing forces, it is crucial that the tested person responds to the applied force. This could be enabled by an isokinetic device if loads would be gradually applied, as performed by Oranchuk et al. (2021) recently [12]. However, different slopes of the force–time curve were suggested in determining the AF during a manual muscle test. These include an exponential phase in the beginning [4]. Another approach is to use pneumatics. A prototype of a pneumatic AF-measuring system (SeBit) has already been constructed and evaluated [3]. During an AF-measurement with the SeBit, a pneumatically driven lever pushes against the limb of the participant. The participant’s task is to resist and hold the given start position for as long as possible. Due to the continuous external force increase, two phases of muscle actions generally occur: firstly, an isometric, and secondly, an eccentric one. During the isometric phase, the participant holds the limb position isometrically by adapting adequately to the increasing external force. Thereby, the maximal isometric AF (AFiso_max_) is determined as the force value at the moment when the tested limb starts to give way. From this moment on, the person is no longer able to hold the starting position isometrically, i.e., its maximal holding capacity is exceeded. Despite this, the external force increases further on, and the participant should still try to adapt to this force increase by decelerating the pneumatically driven lever as strongly as possible (eccentric phase). The highest force value during this eccentric muscle action is referred to as AFecc_max_ [3]. That eccentric maximum normally corresponds to the highest force value of the total AF-measurement (AF_max_). However, AF_max_ can also be achieved during isometric conditions if no eccentric phase exists (voluntary stop of the subject), or if more than one isometric phase occurs.

In measuring the AF, the SeBit showed some methodological limitations (see discussion for further details). Hence, the system was refined and new prototypes for the detection of the AF of the elbow and knee extensors, as well as flexors, were constructed. In this study, only the prototype for the elbow extensors was considered. Thereby, the maximal isometric holding capacity (AFiso_max_) as an expression of the sensorimotor control during the adaptation to increasing external forces was of special interest. Such a force parameter was not measured by other research groups. The main objective was to examine whether or not the AFiso_max_ is a specific and independent parameter of muscle function. To do this, the discriminant validity of the AFiso_max_ was analyzed in comparison to other maximal forces measured by the same system. As a condition of validity, the different forces measured by the new pneumatic device need to be reliable, which was tested in a first step. Subsequently, AFiso_max_, AF_max_ and maximal voluntary isometric contraction (MVIC) were compared and correlated with each other.

As a side question, the influence of a concentric phase prior to an MVIC was analyzed (MVICpri-con vs. MVIC). This specific task of running into an isometric action from a preliminary concentric one could be understood as a functional counterpart of an AF-measurement, which starts with isometry merging into eccentrics. An AF-measurement, as well as the stretch–shortening cycle, were described as a composed muscle action (isometric–eccentric and eccentric–concentric, respectively) [5]. From this point of view, the MVICpri-con could be another variant of a composed muscle action (concentric–isometric).

At last, the MVICs before and after the AF and MVICpri-con measurements were compared (pre-MVIC vs. post-MVIC). A difference might be present because the participants had to perform 12 maximal muscle actions during the whole data collection on one day.

## 2. Materials and Methods

### 2.1. Subjects

To determine the sample size a priori, G-Power (v 3.1.9.3, Düsseldorf, Germany) and a web-based sample size calculator for reliability studies [13], which uses the formula of Bonett [14], were utilized. According to previous studies, very high intraclass correlation coefficients (ICC = 0.920–0.974) were found for MVIC tests (test–retest data of elbow extensors) and AF-measurements (interrater reliability data of knee extensors) [3,15]. Thus, the expected ICC were set at 0.920 for the test–retest design of two sessions in the present study. A minimal sample size of *n* = 11 was calculated to reveal a desired 95% confidence interval (95%CI) of ±0.1 [13,14]. For comparative analyses between force types, a two-tailed *t*-test of differences between two dependent means was chosen in G-power. The *α* and 1–*β* were conventionally set at 0.05 and 0.8, respectively. A minimum of 12 subjects was calculated to detect a substantial effect size of Cohen’s *d_z_* = 0.9, corresponding to a mean difference which is slightly lower than the standard deviation.

In total, 13 healthy Caucasians participated (nine males: 29.38 ± 6.35 yrs., 178.56 ± 4.19 cm, 75.39 ± 9.70 kg and four females: 32 ± 2.94 yrs., 166.75 ± 4.57 cm, 57.00 ± 1.41 kg). The exclusion criteria were any complaints of the upper extremity, spine or head within the last six months. Only the dominant arm was examined. All subjects were right-handed except for two male left-handers. The study was conducted according to the declaration of Helsinki [16], and local ethical permission of the University of Potsdam, approval no. 33/2015, was given. All subjects provided their written informed consent to participate in this study.

### 2.2. Pneumatically Driven Measuring System

**Figure 1:**
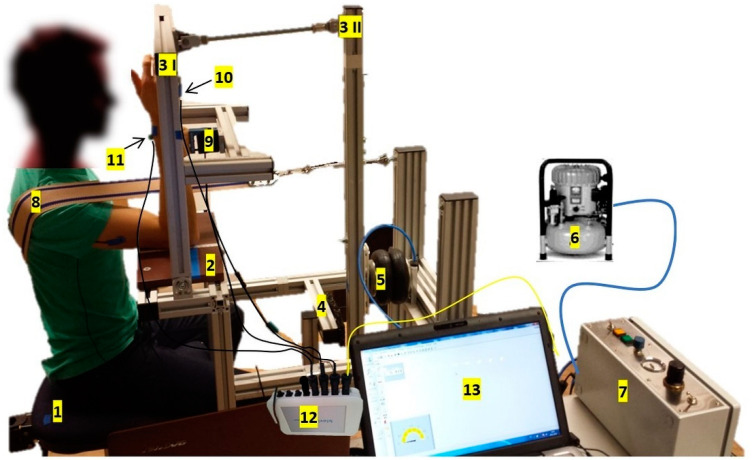
Pneumatic system for the quantification of the AF of the elbow extensors: (**1**) chair, (**2**) table, (**3**) pivoted and connected levers (I and II), (**4**) mechanical security stop, (**5**) frictionless bellows cylinder, (**6**) compressor, (**7**) pressure control unit, (**8**) strap for a dorsal stabilization of the shoulder, (**9**) interface with strain gauge, (**10**) accelerometer I (of lever I), (**11**) accelerometer II (forearm), (**12**) analog to digital converter, (**13**) laptop.

The new measurement system for the assessment of the AF of the elbow extensor muscles is based on the main idea of SeBit [3]. Figure 1 illustrates all components of the new prototype. It enables the subject to adapt to a continuously increasing external force. Newly, the resulting forces and movements of the subject’s arm and the lever I of the device are directly recordable. The generated force of the subject is transmitted to lever I through an interface, which is connected to a strain gauge (force recording, LMZ 2000N 3006, modified by Biovision, Wehrheim, Germany). The interface is lined with cushion to make the force transmission more comfortable. Two accelerometers (modified by Biovision, Wehrheim, Germany) record the movements of the lever I (ACC I) and the forearm (ACC II). The pressure is recorded by a sensor of the control unit. A laptop with the software DIAdem 12.0, National instruments (NI, Austin, TX, USA), receives and saves the amplified signals via an analog to digital converter (ADC) with a sampling rate of 1000 Hz. Table 1 summarizes the main components, the measuring equipment and technical data.

### 2.3. Setting and Procedure

Figure 1 shows the measurement position. The subject’s elbow joint was placed in line with the rotational axis of lever I. The angle between the upper arm and trunk was ~80° to avoid a full contact of extensor muscles with the table. A strap stabilized the shoulder from dorsal. The ulnar side of the distal part of the forearm had contact with the cushioned interface. The interface was in sagittal plane of the shoulder joint (adduction–abduction 0°, internal–external rotation 0°). It was adjusted so that the subject’s forearm was in a vertical position when the lever I was set perpendicular to the table surface (90°). Each subject came for two measuring sessions (t_1_ and t_2_) separated by 7 days. All measurements were performed by following a standardized protocol and the same procedure each day. The procedure was controlled by the same two researchers (first one: operation of the control unit and software; second one: adjustment and supervision of the measurement position). Neither visual feedback nor knowledge of result were given to the subject. For an exact documentation, all measurements were recorded by a video camera.

For a warm up, each subject extended the elbow 20 times against the resistance of an elastic band (Thera-band^®^, level 1 or 2, in dependence of the estimated strength of the subject). Then, four measurement series (a)–(d) were conducted. Prior to each series of (a)–(c), one submaximal trial was executed, so that the subject was able to acclimatize to the referring setting and task. The resting periods were 60 s after series (a) and (b), and 120 s after series (c). Series (b) and (c) took place in a randomized order (coin toss) but the order was identical at t_1_ and t_2_.

(a) Pre-MVIC series

To measure the MVIC, the pneumatic system was inactive, while the subject pushed against the fixed lever I as strongly as possible (pushing isometric muscle action = PIMA). For that, lever I was adjusted to 90°. Every subject was instructed to increase the force up to their maximum within 3 s and sustain this for 1 s. Four trials with resting periods of 60 s were performed.

(b) MVICpri-con series (performed at first in *n* = 7)

Here, again, the pneumatic system was inactive but lever I was not fixed. The subject pushed against the interface while the pressure system was closed. Thereby, lever I slightly gave way in the beginning, with rising resistance due to the increasing air compression, until a steady state was reached at the maximum. Hence, the elbow extensors were firstly activated concentrically and then isometrically. The starting position of lever I (99.58° ± 2.79°) was adapted to the pre-MVIC, so that a steady state was reached at ~90° (mean = 89.80 ± 2.76°). The instructions were identical as in (a). Four trials with resting periods of 60 s were performed.

(c) AF series (performed at first in *n* = 6)

For measuring AF, the pneumatic system was active. Thereby, the pneumatically driven lever II connected to lever I pushed against the subject’s forearm. Lever I was adjusted to 85° in the starting position. In contrast to (a), 5° less elbow flexion was granted to meet the adjustments of the arm in the beginning, which were seen in pre-tests. In the starting position, the subject had a slight contact with the interface (0.62 ± 0.88 Nm ≙ 1.22 ± 1.74% of the pre-MVIC at t_1_). The arm position should be maintained for as long as possible, while the pressure in the system increased over time. For that, the subject had to adapt permanently to the increasing external force in an isometrically holding manner. As soon as the forearm started to give way (isometric muscle actions merged into eccentrics), the subject should still try to decelerate lever I as strongly as possible, until it reached a mechanical security stop at 107° (greatest elbow flexion) or voluntary fatigue. The standardized pressure increase was adjusted manually by a throttle valve in relation to the pre-MVIC, i.e., 70% were reached after 2.5 s (norm under stable conditions). Four trials with resting periods of 120 s were performed.

(d) Post-MVIC series

This measurement series was performed analogous to (a), with only two trials. 

### 2.4. Data Processing 

Data processing was made by the use of the Software NI DIAdem 12.0. The raw data were filtered (lowpass Butterworth, filter order 10, cutoff frequency 3 Hz for force and pressure signals and 1 Hz for ACC signals, respectively, since this provided the most accurate filtering results).

#### 2.4.1. Determination of the Maximal Voluntary Isometric Contractions and the Maximal Adaptive Force

Regarding the measured forces, the maximal values in volts of the pre-MVIC, MVICpri-con, AF_max_ and post-MVIC were determined by the highest value of each trial.

#### 2.4.2. Determination of the Maximal Isometric Adaptive Force

**Figure 2:**
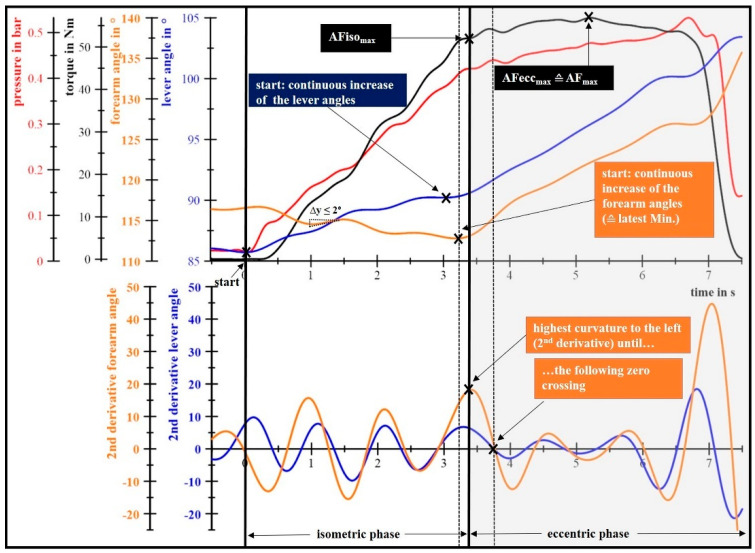
Exemplary AF-measurement of a male person (23 yrs, 1.81 m, 73 kg) to illustrate the algorithm of determining the maximal isometric Adaptive Force (AFiso_max_), with all identified points (x) in filtered curves (lowpass Butterworth, filter order 10, cutoff frequency 3 Hz for torque (black) and pressure (red), or 1 Hz for lever angle (blue) and forearm angle (orange)).

The determination of AFiso_max_ needs a more sophisticated approach. AFiso_max_ corresponds to the highest force value at the moment when the forearm starts to give way for the first time. A standardized algorithm was utilized to identify this timepoint (Figure 2). For that, the recorded volt signals of the ACC sensors were converted into angles (ACC I for the lever angles and ACC II for the forearm angles). It should be noticed, that the “angles of the forearm” do not reflect the elbow angle. Due to adjustments of the arm position in the beginning of the trial, intermittent deviations of ≤2° of the forearm angles were tolerated and still interpreted as isometric muscle actions.

The start of giving way was not always as clear as in Figure 2 and could not be determined easily as the start of a continuous increase in one of the angle curves (latest minimum within the determined 2°-tolerance). That is because, in some measurements, only a very slight increase was present before a considerable “break-off” (steep increase) appeared. Thus, the second derivative of the angle curves was calculated to use information about their curvatures. Referring to the considerable break-off, the start of giving way was defined as the timepoint of the highest curvature to the left, directly after the latest minimum of the time–angle curves (start of the continuous increase of forearm or lever I) and before the following zero crossing in the second derivative. In dependence of the latest minimum (angle curve of forearm or lever I), the respective second derivative was used. Differences between the forearm and lever I angle curves appeared due to filtering effects and shifts of the elbow, especially to the posterior, which could not be avoided completely by the dorsal shoulder strap. Furthermore, the back-shift can partly explain the initial decrease in the forearm angles of about 4° in Figure 2. In contrast, the lever I angles increased initially (~5°), since lever I was driven by the subject who had only a slight contact with the cushion of the interface, which was therefore crumpled. Due to this crumple zone, a decrease in forearm angles was also seen in MVIC measurements.

An AFiso_max_ during a pushback of the lever I of ≥ 0.3° was defined as task failure (pushing the lever backwards instead of holding it in position). It resulted in an exclusion, since the subject then switched into a PIMA [9]. That concerned 1 out of 104 AF-measurements in the present study. The strict limit of 0.3° was chosen according to pre-tests with PIMAs.

The described algorithm was proven on > 800 AF-measurements (elbow and knee extension, as well as flexion). The determined AFiso_max_ values correspond optically to the break-off point in 99.53%.

#### 2.4.3. Determination of the Maximal Eccentric Adaptive Force

The AFecc_max_ was determined as the highest force value during the eccentric phase, which started usually after AFiso_max_. It was ended as soon as lever I hit the mechanical security stop (termination of the measurement) or, if present, during the start of another isometric phase. AFecc_max_ was excluded from statistical analyses because it was identical to AF_max_ in 98 of 104 measurements. In 3 measurements AFecc_max_ could not be determined because AFiso_max_ = AF_max_, whereby AFecc_max_ could not be detected (no eccentric phase due to a voluntary stop of the subject). The 3 remaining AFecc_max_ values were only slightly different from AF_max_ (mean difference = 0.41 ± 0.59 Nm). 

#### 2.4.4. Calculation of Mean and Maximal Torques and Elapsed Times

All force values (V) were converted into N (conversion factor: 124.74 $\frac{N}{V}$). For comparability, torques in Nm were calculated (T = F × r), whereby F was the force in N and r was the individual distance of the rotational axis and the middle of the interface in dependance of the forearm length. The arithmetic mean (M) and maximal (Max) torques out of the single trials of each measurement series were determined as the main variables of interest. The minimal, maximal and mean elapsed times (±standard deviation (SD)) from start to AFiso_max_ and AF_max_ were calculated for all trials and subjects.

### 2.5. Statistical Analyses 

For the statistical analyses, IBM SPSS Statistics 26 was used. The M and Max torques of pre-MVIC, post-MVIC, MVICpri-con, AFiso_max_ and AF_max_ were considered. All M and Max torques of the total sample (*n* = 13) were normally distributed (Shapiro–Wilk test, *p* > 0.05). Thus, parametric tests were used. Due to deviations from normal distribution, differences between males (*n* = 9) and females (*n* = 4) were tested by the Mann–Whitney *U* test.

For all tests regarding reliability, a liberal significance level of *p* = 0.10 was used, recommended by Weir et al. (2005) [17]. In regard of all other analyses, the conventional *p* = 0.05 was chosen. For significant results of parametric tests, effect sizes were calculated by Cohen’s *d_z_*:

(1) $$d_{z}=\frac{\left|MD\right|}{SD_{MD}},$$

whereby *MD* is the mean difference between t_1_ and t_2_ and *SD_MD_* its standard deviation. According to Cohen [18], the effect sizes were interpreted as small, moderate or large (<0.50, 0.50–0.80, ≥0.80, respectively). In non-parametric tests, the effects sizes were calculated by Pearson’s *r*:

(2) $$r=\sqrt{\frac{Z}{N}}$$

It was interpreted as being small (*r* = 0.1–0.3), moderate (*r* = 0.3–0.5) or large (*r* ≥ 0.5) [18].

#### 2.5.1. Reliability

A reliability analysis was performed mainly according to the suggestions of Atkinson and Nevill (1998) [19]. In a first step, the presence of a systematic change between t_1_ and t_2_ was tested by a paired *t*-test. The 90% confidence intervals (90%-CI) are presented.

To decide between an absolute or relative quantification of the measurement error, the scedasticity of M and Max torques was evaluated in two ways: graphically by scatter plots (absolute difference between t_1_ and t_2_ against the individual measurement mean) and statistically by the Breusch–Pagan test for heteroscedasticity of the standardized residuals in a linear regression (t_1_ vs. t_2_). Deviating from that in one exception, the White test was used for the M of AFiso_max_ because the standardized residuals in the regression analysis differed from normal distribution (Shapiro–Wilk test, *p* > 0.05). The standardized residuals of all other variables were normally distributed. According to the statistical analysis, every variable should be interpreted to be homoscedastic, since the variances of the standardized residuals were equal across the whole continuum of torques (*p* = 0.061–0.985, the complete statistics of the tests for heteroscedasticity can be found in Figures S1 and S2 in the supplementary material.) Thus, absolute reliability was quantified by the standard error of measurements between t_1_ and t_2_ (SEM), which is also known as the within-subject variation [20]. It is expressed by using the generalizability approach [21]:

(3) $$\mathrm{SEM}=\sqrt{\sigma_{e}^{\;2}},$$

whereby σ*_e_*^2^ is the variance of the random error extracted from the residual variance of the repeated measures ANOVA [22,23]. For the practical relevance of individual measurements, the minimal detectable change, which is also known as the smallest detectable difference, was calculated by [17,21,22,24,25]:

(4) $$\mathrm{MDC}95\%=1.96\times SEM\times \sqrt{2}.$$

However, homoscedastic data are very uncommon in ratio scales as strength measures [26]. Furthermore the scatter plots partly show increasing absolute differences between days by increasing means (= positive heteroscedasticity) (see Figures S1 and S2 in the supplementary material). A logarithmic transformation of those data, as suggested by some authors [26,27], would not result in sufficient homogenization. Thus, random errors (SEM) were also presented in relation to the respective group mean of t_1_ and t_2_ (*M*_*t*_1_,*t*_2__) (percentage error) to take the positive heteroscedasticity into account [19]:

(5) $$\mathrm{SEM}\%=\frac{SEM}{M_{t_{1},t_{2}}}\times 100.$$

Similar to the MDC95%, the SEM95% was given and expressed as the SEM% multiplied with the *z*-score of 1.96 [19].

The relative reliability of M and Max torques between t_1_ and t_2_ was quantified by the intraclass correlation coefficient (ICC(3,1) (two-way mixed, absolute agreement, single values), which is unbiased for any sample size [20]. Additionally, 95%-CI were calculated for the ICCs.

#### 2.5.2. Discriminant Validity of the Maximal Isometric Adaptive Force

The AFiso_max_, AF_max_ and pre-MVIC (M and Max torques) were compared with each other by a paired *t*-test. To analyze relations between the two AF parameters and the pre-MVIC, Pearson’s correlation coefficients were calculated.

The AF parameters of all single trials were normalized to the M and Max of the pre-MVIC. The normalized data were presented as M ± SD in % and were compared by Wilcoxon signed-rank test because the normalized values were not normally distributed. In addition to the normalization to the pre-MVIC, the AFiso_max_ of each single trial was normalized to the respective AF_max_.

The variability between the 4 trials of a measurement series was expressed as M ± SD of individual coefficients of variation (CV) for each day.

#### 2.5.3. Analyses Regarding the MVIC with a Prior Concentric Contraction and the Post-MVIC

For the last two analyses, paired *t*-tests were used. To analyze the influence of a concentric phase prior to the MVIC, M and Max of MVICpri-con were compared with the respective ones of pre-MVIC. At last, the M and Max torques were compared between the pre- and post-MVIC.

## 3. Results

### 3.1. Gender Comparison

M and Max (± SD) torques of all measurement series at both days are presented in Table 2. Males revealed significantly higher torques of the same force type than females, on average (*z* = −2.777–−2.623, *p* = 0.005–0.009, *r* = 0.45–0.46). Torques of all single trials and force types can be found in Table S1 in the supplementary material.

### 3.2. Description of AF-Measurements

Figure 2 exemplifies the typical curves of one AF-measurement. During their adaptation to the pressure increase (red), a subject’s generated torque (black) increased over time. Due to the compression of the cushion between the subject’s forearm and the interface, as well as little back-shifts of the elbow, an increase in lever angle (blue) and a decrease in forearm angle (orange) occur initially. A deceleration of the lever and further decrease of the forearm angles interrupted by slight oscillations within the 2°-tolerance indicate that the forearm was held in position. As soon as the forearm starts to give way, the AFiso_max_ is exceeded and the eccentric muscle work begins. Thereby, the pressure and torque curves increase further on with a flatter slope, until the highest torque is reached (AF_max_ = AFecc_max_).

The mean elapsed time until AFiso_max_ over all measurements was 2.78 ± 0.94 s (Min.–Max.: 0–4.50 s). AF_max_ was reached after 4.57 ± 0.79 s (Min.–Max.: 3.21–6.87 s). 

### 3.3. Test–Retest Reliability

**Figure 3:**
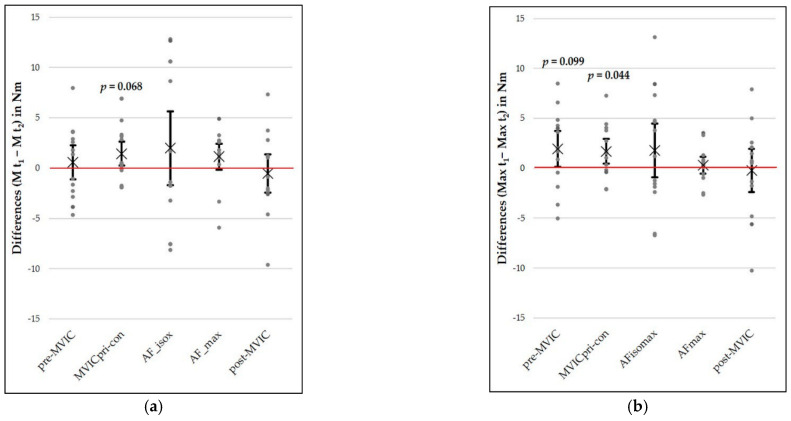
Differences between day 1 (t_1_) and day 2 (t_2_) for mean (M) (**a**) and maximal (Max) torques (**b**) out of 4 measurements of all measurement series. Dots illustrate single values. The mean difference is marked by “×”. Error bars express 90% confidence intervals.

Figure 3 illustrates the group mean (“×”) and individual differences (dots) between t_1_ and t_2_ for M, as well as Max torques of all force types. The Max of pre-MVIC (*p* = 0.099), as well as the M (*p* = 0.068) and Max (*p* = 0.044) of MVICpri-con, differed significantly between t_1_ and t_2_. Thereby, the torques at t_2_ were consistently lower than those at t_1_. The effect sizes were *d_z_* = 0.52, 0.56 and 0.62, respectively. All other between-days comparisons differed insignificantly (*p* = 0.175–0.869). The complete inference statistics, SEMs, MDC95%s, SEM%s, SEM95%s and ICCs(3,1) with 95%-CIs are given in Table 3. The highest occurred mean difference amounted to 1.98 ± 8.03 Nm (M AFiso_max_). The SEMs, MDC95%s, SEM%s and SEM95% ranged from 1.29 to 5.68 Nm, 2.53 to 15.70 Nm and 2.53 to 15.70%, respectively, where the highest values occurred in AFiso_max_. All ICCs were greater than 0.89 (Table 3). 

### 3.4. Comparisons of Force Types

#### 3.4.1. Comparison between AF-Parameters and the Maximal Voluntary Isometric Contraction

**Figure 4:**
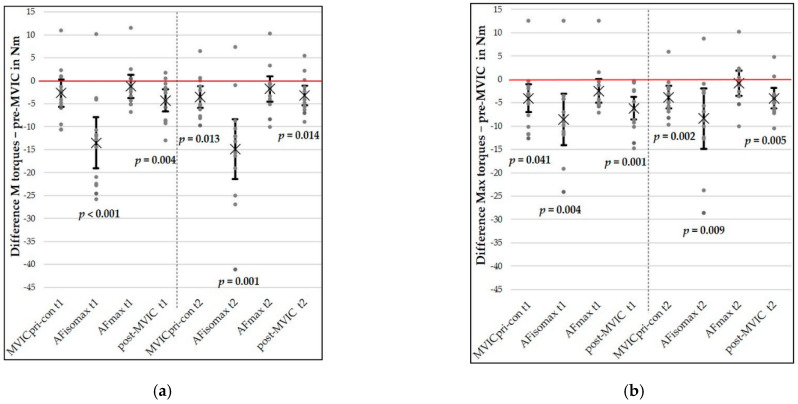
Comparisons of the pre-MVIC and all other measured force types on day 1 and day 2 (t_1_ and t_2_). Mean (M) (**a**) and maximal (Max) torques (**b**) of the pre-MVIC are subtracted from the respective value of other force types). Dots illustrate single values. The mean difference is marked by “×”. Error bars express 95% confidence intervals.

Figure 4 shows the mean and individual differences for M and Max torques between the pre-MVIC and all other force types, as well as between AF_max_ and AFiso_max_ on both days. No significant differences were found between AF_max_ and pre-MVIC neither at t_1_ nor at t_2_ (M and Max: *p* = 0.109–0.531). The complete inference statistics can be found in Table 4. M and Max torques of AFiso_max_ were significantly lower than those of the pre-MVIC, as well as those of AF_max_ on both days (*p* ≤ 0.001–0.009 and *p* ≤ 0.001–0.002, respectively) (see Table 4). 

The correlations between AFiso_max_, AF_max_ and pre-MVIC at t_1_ and t_2_ ranged from *r* = 0.85 to 0.98. All correlations were significant (*p* ≤ 0.001). Correlations which involved the AFiso_max_ were lower (*r* = 0.85–0.97) than those between AF_max_ and pre-MVIC (*r* = 0.97–0.98).

**Figure 5:**
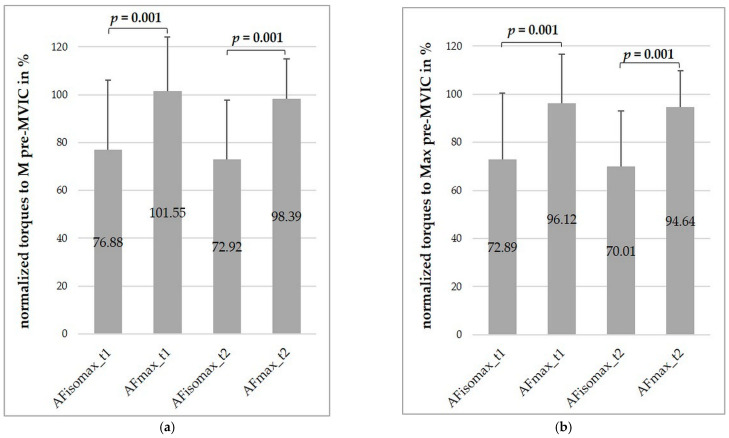
Comparisons of the average maximal isometric Adaptive Force (AFiso_max_) and average maximal Adaptive Force (AF_max_), normalized to the mean (M) (**a**) and maximal (Max) torques (**b**) of the pre-MVIC on both days (t_1_ and t_2_). Error bars express between-subject standard deviations.

The averages of the normalized AFiso_max_ and AF_max_ are presented in Figure 5 (normalized to the M pre-MVIC (a) and to the Max pre-MVIC (b)). The normalized AFiso_max_ was significantly lower than the normalized AF_max_ on both days (each *z* = −3.18, each *p* = 0.001, each *r* = 0.5). The AFiso_max_ normalized to the AF_max_ amounted to 74.64 ± 14.51% at t_1_ and 72.92 ± 15.72% at t_2_.

The CVs between single trials of AFiso_max_ were substantially greater (t_1_: 28.65 ± 21.95%, t_2_: 24.92 ± 18.89%) compared to those of pre-MVIC (t_1_: 5.32 ± 3.04%, t_2_: 4.16 ± 3.01%), AF_max_ (t_1_: 3.25 ± 1.72%, t_2_: 5.40 ± 2.92%) and MVICpri-con (t_1_: 2.99 ± 1.94%, t_2_: 3.32 ± 2.21%).

#### 3.4.2. Comparisons between Measurement Series Including a Maximal Voluntary Isometric Contraction

In regard to the comparisons of pre-MVIC and MVICpri-con, M torques at t_2_, as well as Max torques at t_1_ and t_2_, differed significantly (*p* = 0.013, 0.041 and 0.005, respectively, Table 4). Thereby, MVICpri-con showed lower torques. Effect sizes were 0.81, 0.64 and 0.94, respectively. No significant differences were found between M torques at t_1_ (*p* = 0.103).

M and Max torques of post-MVIC were significantly lower than those of pre-MVIC at both sessions, (*p* = 0.001–0.014, *d_z_* = 0.80–1.26, Table 4).

## 4. Discussion

In the first step, the methodological quality of the new measurement procedure will be considered as a prerequisite for the following discussion. That also includes the advantages over SeBit and its limitations. Subsequently, the influence of a concentric contraction prior to MVIC, as well as possible fatiguing effects, will be discussed. The integration of the AF into current concepts of human strength and the specialty of the AFiso_max_ will be our focus at last.

### 4.1. Reliability of the Measured Forces

The comparison of M and Max torques between days revealed no significant differences in most force types. Thus, a systematic change between days in AFiso_max_, AF_max_ and post-MVIC is not assumed. In contrast, the Max of pre-MVIC, as well as M and Max of the MVICpri-con, differed significantly between t_1_ and t_2_ (*p* < 0.1, liberal α-level). Thereby, the torques were always lower on average at t_2_, whereby moderate effect sizes occurred (*d_z_* = 0.50–0.62). An unlikely effect of insufficient regeneration after 7 days of rest cannot be ruled out completely. However, that would raise the question why it was not seen in the other force variables. Several authors suggested adapting the measurement protocol and examining the reliability again if systematic changes are evident [17,19]. This might be considered, especially for the unconventional MVICpri-con measurements. The MVIC of elbow extensors measured by strain gauge had already proven to be reliable [15]. Furthermore, the difference between t_1_ and t_2_ of the Max pre-MVIC was very close to insignificance (*p* = 0.099). However, results of paired *t*-tests as sole reliability statistics are not recommended because the detection of systematic changes depends highly on random errors [19]. A significant result in a *t*-test could be explained by two approaches. Either the true variance is very high or the random error is very small. Inversely, an insignificant result can be explained by a high random error.

The random errors between t_1_ and t_2_, which cover 65% of all measurements, are provided as SEMs in the unit of interest (Nm) and as a percentage (SEM%) (Table 3). The latter is a type of within-subject coefficient of variation (CV) but calculated from the mean square error term in a repeated-measures ANOVA model, as suggested by Atkinson and Nevill (1998) [19]. CVs of ≤10–15% are conventionally rated as being reliable in sport science [28,29,30]. However, those thresholds are also criticized [19]. Independently of the proposed threshold (≤10% or ≤15%), only the means of AFiso_max_ would exceed it (15.70%). All other force variables revealed lower CVs between days (2.53–9.43%), whereby the Max of AFiso_max_ showed the highest. The higher random errors between days (variability) in AFiso_max_ might be explained by the specialty of this force type, which will be discussed in a later subsection.

Rather than rating the results as reliable or not according to a CV, it is more important to consider the presented random errors for the interpretation of future interventional studies. In dependence of the assumed scedasticity, the SEMs or CVs could be used for prospective group comparisons. In contrast, to compare changes of an average individual, MDC95%s or CV95%s should be used, at least because these would cover 95% of the repeated measurements [19]. That means exemplary for Max torques of AFiso_max_, group changes ≤4.17 Nm (SEM) and individual changes ≤11.56 Nm (MDC95%) are within the area of measurement error and should not be declared as relevant, despite possible significant differences. Absolute errors (SEM and MDC95%) are used because, in the presented study, Max AFiso_max_ data are rated as homoscedastic (Figure S2 in the supplementary material).

To the authors’ best knowledge, the study provided first data of MVICpri-con, AFiso_max_ and AF_max_ of the elbow extensor muscles. Thus, the random errors cannot be compared with other studies. Concerning the MVIC, Meldrum et al. (2003) presented SDs of the mean differences (Max values out of two trials) of the elbow extensors captured by a strain gauge [15]. In experienced raters, the SD_MD_ amounted to ±1.46 kg (≙a SEM of $\frac{1.46\;\mathrm{kg}}{\surd 2}$ = 1.03 kg) for the left and ±1.58 kg (≙ SEM of 1.12 kg) for the right arm. The formula for the calculation of the SEM is similar to the one we used [17]. These SEMs are close to our presented SEMs of Max torques (converted to kg ≈ 1.15). This comparison should be interpreted with care because of methodological differences between the studies. The point of force application was also proximal of the wrist, but the measurement position differed compared to our study (supine vs. seated).

Relative reliability is commonly interpreted by the ICC [31]. It reflects reliability in relation to a measured sample. According to Koo and Li [32], the presented ICCs were good to excellent. The lowest, but still in good agreement, was found for mean values of AFiso_max_ (ICC = 0.896), whereas Max AFiso_max_ achieved an excellent ICC of 0.956. The ICCs of all other variables were also excellent (0.976–0.996). Regarding the MVIC, Meldrum et al. (2003) also reported excellent ICCs (0.92–0.95) of M and Max values for the left and right elbow extensors [15].

In summary, all measured force types in the presented study, and especially the introduced AF parameters, are interpreted as revealing sufficient reliability.

### 4.2. Advantages and Limitations of the New AF-Measurement Procedure

The introduced AF device for the elbow extensors is a refinement of the SeBit. Both devices can measure the MVIC, the MVICpri-con and the AF parameters [3]. The SeBit only provided pressure signals. In contrast, the new device uses a strain gauge to record force and ACCs to detect the movements of the limb and lever. As a consequence, AF_max_ is always detectable and AFiso_max_ is easier to identify without an analysis of the deviation from a reference curve, as was done before [3]. Moreover, the frictionless bellows cylinder eliminated the stick–slip effect which occurred in the former used cylinder, including a push rod [3]. Another advantage over the SeBit is the individualized pressure increase based on the MVIC of a subject. Due to these advances, the assessment and determination of the AFiso_max_ is more standardized and accurate with regard to a subjects’ properties.

However, some limitations of the new system have to be pointed out. For individualization, the pressure increase was adjusted so that the external force would reach 70% of the MVIC after 2.5 s. The actual duration was 2.90 ± 0.84 s. The pressure increase depends not only on the incoming air but also on the extension of the bellows cylinder. If the cylinder expands, the pressure, and consequently the force increase, will flatten, i.e., the pressure course would only be fully standardized if the subject holds the lever in a completely stable position. That depends on the subject’s ability to hold the lever in position. However, even in measurements with a high AFiso_max,_ a lever drive occurred, as described before. Consequently, slightly different pressure increases occurred. However, the elapsed time until AF_max_ was 4.57 ± 0.79 s. From our point of view, this duration is reasonable, but the optimal pressure increase for capturing the AF needs to be discussed and examined in future studies [4].

Another limitation is related to the characteristics of the initial pressure course. A sudden increase was evident, especially in subjects with high MVICs. The pressure increase, according to the standardization of reaching 70% of the MVIC after 2.5 s, was relatively steep for those subjects, and the start might have been abrupt. Independent of the MVIC, a general smooth start through a motor-controlled valve will solve this problem in the next generation of the system.

As a further limitation, the determination of AFiso_max_ has to be considered. In the described algorithm, the start of giving way of the forearm up to a boundary of 2° was tolerated and interpreted as an isometric holding phase (quasi-isometric muscle action). Setting a boundary was necessary because complete isometrics in a nearly freely oscillating system is not possible, and because of a slight elbow shift, which occurred in all subjects at the beginning of the AF-measurements. It reflects an initial adjustment of the subject and a compression in the respective joints during the isometric phase. It could not be avoided, although the fixation of the subject’s arm and shoulder was already strong. A complete fixation is not possible. However, higher or lower boundaries (instead of 2°) would change the AFiso_max_. This especially plays a role in measurements with two or more isometric phases, which occurred in 7 of 104 measurements. The current algorithm considers only the first isometric phase. Furthermore, flat slopes in lever angles (slowly giving way) were not included in the isometric phase (31 measurements). Both a second isometric phase and flat slopes characterize a different quality of resisting an external force compared to a steep incline in angles. An influence of the determination method on reliability statistics cannot be excluded completely. However, based on the high agreement (99.53%), with the break-off point in > 800 measurements, the algorithm already seems to be quite sophisticated.

### 4.3. Influence of a Concentric Muscle Action Prior to a Maximal Voluntary Isometric Contraction

The MVICpri-con firstly includes a concentric muscle action which merges into an isometric one [3]. Thereby, the force maximum is always reached during isometrics.

In the presented study, the comparison of MVICpri-con and pre-MVIC revealed inconsistent results. No significant difference of the M torques at t_1_ was found. In contrast, M torques at t_2_ and Max torques at both days were significantly lower than the respective pre-MVICs torques, with moderate to large effect sizes (0.64–0.94). In both measurement series, the force values were reached at similar lever angles (90° vs. ≈ 89°). Thus, differences in muscle length can be excluded as a reason for the lower force in MVICpri-con. The duration until reaching the MVICpri-con values lasted 0.5 s longer than reaching the pre-MVICs (4.57 ± 0.92 s vs. 4.07 ± 0.83 s), which could serve as an explanation. However, the lower force could rather be explained by the order of measurements. Six subjects performed the MVICpri-con series after the AF series. If only the other seven subjects who performed the MVICpri-con series first had been considered, no significant differences would have been found (*p* = 0.175–0.602). Due to the lower sample size, this should be interpreted with caution. Possible underlying fatiguing effects are discussed in the next section.

The results suggest a prior concentric phase might not have a clear influence on the MVIC, but more research is necessary for a final conclusion. Moreover, the potential systematic biases of MVICpri-con measurements are already mentioned in the reliability section above. Future studies could adapt the measurement protocol by examining only these two forces in a randomized order.

### 4.4. Comparison of MVICs at the Beginning and at the End of Each Measurement Session

The M and Max torques of post-MVIC (performed after 12 other maximal muscle actions) were significantly lower than the respective pre-MVIC at both days, with large effect sizes (*d_z_* = 0.80–1.26). This could be an indication of potential central and/or peripheral fatiguing effects. On the one hand, the neuromuscular system could have been already neurologically and/or structurally exhausted, especially because of the usually involved eccentric loading during the AF-measurement [33,34,35]. In this regard, a reflectory inhibition of motoneurons might also play a role. Other reasons could be mental fatigue or a lack of motivation. The motivation to activate the muscles maximally might have declined towards the end of the session. In future, fewer measurements and/or measurement series might eliminate possible fatiguing and/or motivational effects.

### 4.5. Integration of the Adaptive Force in Current Concepts of Strength

The determination of MVIC is the gold standard for investigating maximal isometric strength, which is one central parameter in strength diagnostics [36,37,38,39,40]. Comparisons and correlations of the pre-MVIC with the recently introduced AF parameters were made to provide information concerning their discriminant validity. All respective variables (M and Max torques) revealed a sufficient reliability (see subsection about reliability).

In respect of the comparison of pre-MVIC and AF_max_, the M and Max torques did not differ significantly. Nearly all AF_max_ values occurred during the eccentric phase. Only in three measurements, the AFecc_max_ was somewhat lower than AF_max_ (mean difference = 0.41 ± 0.59 Nm). In the past, significantly higher forces were found during eccentric muscle actions compared to isometric ones [33,41,42,43]. However, it seems that it is not a general phenomenon [44,45,46]. Fitness level [47], examined musculature [48], joint angle or angular velocity [5,44,48,49] are discussed as influencing factors. For example, other research groups which used isokinetic devices reported higher maximal eccentric forces, with higher angular velocities in well-trained subjects [47,50]. Compared to isokinetic measurements, an AF-measurement, as conducted in the presented study, is a completely different approach to measure eccentric forces. It includes quasi-isometric and, afterwards, very slow eccentric muscle work. Thereby, the angular velocity depends on a subject’s ability to decelerate the movement of the lever, which cannot be—and should not be—standardized. The average angular velocity of lever I during all eccentric phases was 2.31 ± 0.50 °/s. The highest value in one single trial was 5.93 °/s. These slow and subject controlled eccentric movements could be an explanatory approach as to why the AFecc_max_ or AF_max_ did not exceed pre-MVIC. In regard to the specific measurement procedure, it is suggested the AF_max_ could be used to assess the maximal force capacity comparable to the MVIC. However, using a more explosive pressure increase and, therefore, higher angular velocity would probably change the result. This was shown in a previous study, whereby higher explosive AF_max_ values measured by the SeBit were found compared to the MVIC [5].

In contrast to AF_max_, the M and Max torques of AFiso_max_ differed significantly (*p* ≤ 0.001–0.009) from those of pre-MVIC at both days, with large effect sizes (*d_z_* = 0.86–1.32). Thereby, AFiso_max_ ranged from 70.01% to 76.88% of the pre-MVIC (SD = ±23.00 − 29.24%). Furthermore, the AFiso_max_ normalized to the pre-MVIC was significantly lower (70.01–76.88% than the normalized AF_max_ (96.12–101.55%, *p* = 0.001). The effect sizes were interpreted as large (*r* = 0.5).

Our research group already suggested considering the AFiso_max_ as a measure of a special neuromuscular function [2,3]. It reflects maximal holding capacity while adapting to increasing external forces, whereas the MVIC declares the maximal isometric contraction in a pushing manner. AFiso_max_ (adaptive HIMA) seems to reveal substantially lower forces than MVIC (PIMA), i.e., the forearm started to give way (muscle lengthening) before the individual MVIC was reached. This confirms the hypothesis of a differentiation between isometric muscle actions in at least two modes (HIMA vs. PIMA) [9]. A difference between HIMA and PIMA was identified concerning, e.g., the time to task failure [9,51]. However, another research group could only confirm this finding in part [6,7]. A HIMA always includes an adaptational component of muscle function. It is suggested that an adaptation to increasing or varying forces is more sensitive to detect differences from a PIMA compared to an adaptation to constant forces. This suggestion is supported by the results of the presented study, revealing a significant difference between AFiso_max_ and pre-MVIC. AFiso_max_ reflects a HIMA, since the subject has to adapt to an increasing force in an isometric holding manner. The MVIC, in turn, characterizes a PIMA, as the subject has to push isometrically against a stable resistance. The cause for the found difference might lie in the higher adaptational component during AFiso compared to the MVIC. As mentioned above, previous studies only found a difference in the time to task failure of PIMA and HIMA with constant forces [6,7,9,51]. The adaptational component was not considered. This emphasizes the importance of determining AFiso_max_ as a special and individual force capability (see below). The investigation could be performed by using the presented measurement system or, in a more practical way, by using a handheld device in combination with a manual muscle test, whereby reproduceable force applications are necessary [4].

Despite partial significant differences, high to very high relationships between the AFiso_max_, AF_max_ and pre-MVIC were found in the present study (*r* = 0.85–0.98). This indicates the AF can be integrated in the conditional ability of strength in healthy subjects. Different research groups found that several force measurements or strength tests correlate with each other, and confirm the hypothesis of generality [52,53,54]. However, the determination coefficients (r^2^) are often lower than 0.5, i.e., <50% of the variances are explained by each other. Such findings question the interpretation of generalization [55]. In the presented study, the determination coefficients between AF_max_ and pre-MVIC were very high (r^2^ = 94.09–96.04%). In contrast, the coefficients regarding AFiso_max_ were lower (r^2^ = 72.25–94.09%). That means the AFiso_max_ cannot be explained by the other two force types as well as they can explain each other.

All of the results regarding the AFiso_max_ indicate that it could be discriminated from the MVIC and AF_max_. Thus, it should be detected separately and could reflect an independent force capability. It is assumed that AFiso_max_ depends not only on the maximal strength of a person. Due to the adaptive component, the influence of a proper functioning of sensorimotor control might play a decisive factor in defining AFiso_max_.

### 4.6. Specialty of the Maximal Isometric Adaptive Force

Adaptation to external forces in an isometric holding manner is a rarely considered motor task. Commonly used strength tests are not able to capture this function. As stated before, the maximal adaptive holding capacity of a muscle can be quantified during an AF-measurement (AFiso_max_). An intact holding isometric muscle function during adaptation to external forces (high AFiso_max_) might prevent an inappropriate muscle lengthening up to considerably high intensities close to its maximal capacity. In the past, animal models indicate that an externally induced lengthening of a tensioned muscle (eccentric muscle action) is the primary injury mechanism of muscle strains [56]. In high speed treadmill and overground running of humans, a muscle strain most likely occurs during the late swing phase, which is accompanied by an eccentric [56], or, from a newer point of view, an isomeric muscle action [57]. According to van Hooren and Bosch (2017) [57], the presence of an inefficient eccentric muscle action in the late swing phase is caused by the inability of the muscle fascicles to act isometrically. Consequently, the muscle is more vulnerable to injury [57]. Moreover, an unwanted muscle lengthening could also lead to an exceeded joint movement in a specific direction, which might cause a traumatic injury, e.g., regarding the anterior cruciate ligament [58], ankle sprains [59] or shoulder dislocations [60]. High susceptibility to injuries during the lengthening of muscles emphasizes the importance of an adequate isometric holding function during adaptation to external forces. In this regard, high isometric holding capacities of muscles (high AFiso_max_) might prevent or at least delay the moment of their lengthening, and consequently stabilize joints up to a higher force level. This is essential, especially in powerful movements or high impacting loads. As one single moment of the muscle’s inability to remain acting isometrically could be harmful, not only the highest AFiso_max_ of several trials but also the minimal value and its variation might be of diagnostic interest.

**Figure 6:**
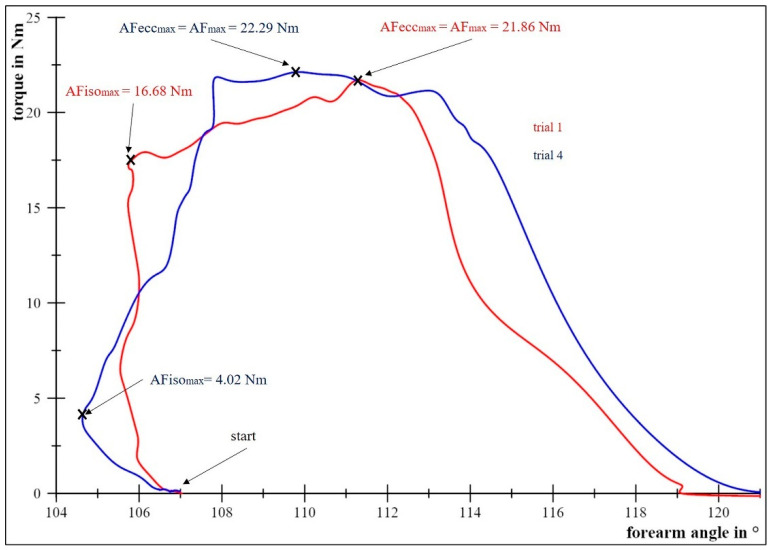
Angle-force plot of two exemplary AF-measurements of the same subject (female, 32 yrs, 1.68 m, 58 kg). In trial 1 (red curve), the arm angle does not change meaningfully until the maximal isometric Adaptive Force (AFiso_max_ = 16.68 Nm) is reached. In trial 4 (blue curve), the forearm starts to give way at a lower force level (lower AFiso_max_ = 4.02 Nm). The maximal Adaptive Forces (AF_max_), which are reached during the eccentric phases (= AFecc_max_), are similar in both trials (22.29 Nm and 21.86 Nm). These two trials illustrate the variability of AFiso_max_.

The present study showed that, even in subjects without health complaints, the forearm started to give way before the maximal isometric pushing force (Max pre-MVIC) was reached. There was only one subject whose AFiso_max_ was greater than the Max pre-MVIC in all trials (+7.88–74.39%). In 11 other trials of six different subjects, AFiso_max_ was greater than 90% of Max pre-MVIC. This demonstrates a high relative AFiso_max_ is possible. Such a high AFiso_max_ is considered as perfect adaptation, as long as the Max pre-MVIC is rated as sufficiently high. Regarding the average of all measurements, the AFiso_max_ amounted to about 70% of the Max pre-MVIC. That percentage was reached by 11 of 13 subjects in at least one single trial (57 in total). However, two subjects never reached that value. Furthermore, in 21 trials of nine different subjects, the AFiso_max_ was even lower than 50% of the Max pre-MVIC. This, in turn, emphasizes the need for an individual analyzation. It also means that lower and higher AFiso_max_ values can occur in different trials of the same subject. This is exemplified by Figure 6. In the blue curve, the start of giving way was at a substantially lower torque (4.02 Nm) compared to the red curve (16.68 Nm). Even if the arm movement could be decelerated up to 22 Nm (blue curve), the determined 2°-tolerance in angles was exceeded. Thus, the muscle action was no longer rated as isometric. Different AFiso_max_ levels in the same subject result in a higher variation between single trials and between days. As described before, AFiso_max_ revealed higher SEMs (M: 5.68 Nm; Max: 4.17 Nm) compared to other maximal forces (SEM = 1.29–3.16 Nm). The variation between single trials was also higher (CVs ≥ 24.29% vs. ≤ 5.40%). The greater within- and between-days variations imply a suspected lower reliability of AFiso_max_ compared to other force types. Although the already mentioned limitations in the determination method of AFiso_max_ might play a role (inter alia, strict 2° boundary), these results could be explained from another point of view: We suggest AFiso_max_ has a higher biological variability compared to other maximal forces. It could possibly be present due to a required higher complexity of sensorimotor control during the adaptation of muscular tension and length to varying external forces compared to pushing actions, such as the MVIC test.

The adaptation during a holding action requires an adjustment of the muscular tension, together with the muscular length. The change in muscle length is sensed by muscle spindles and the change in tension by Golgi tendon receptors [61]. The kinesthetic afferences are sent to spinal and supraspinal areas, where they are integrated and processed before an adequate response is performed. These complex feedback control mechanisms for adjusting tension and length have to function properly during an AF-measurement [62,63]. To hold a position over time, the muscular tension and length have to be adjusted immediately after a reference error of the position has been detected. That procedure has to be repeated consecutively during the whole measurement process. However, feedback control mechanisms alone cannot be responsible for maintaining the position because the external force increases over time and a compensatory response to it would always be delayed. Additionally, to deal with this time problem, an adequate feedforward control is required, whereby the force increase must be anticipated [4]. The anticipation must be continuously adjusted on the basis of proprioceptive inputs. This results in further neurophysiological demands, leading to an approximated control of the position which anticipates the forthcoming increase in the external force. Thereby, marginal lengthenings and shortenings of the muscle fibers occur. As the resulting oscillations of the limb are stationary, the muscle action can still be considered as isometric or, more precisely, “quasi-isometric”.

In contrast, by performing an MVIC test (pushing isometric task), the subject must only be proactive and change muscular tension without the need to respond to an external varying force or control the length. Thus, neuromuscular demands ought to be lower compared to an AF-measurement, and might explain why the pre-MVIC was higher and less variable than the AFiso_max_. Compared to a pushing isometric mode, it was already assumed that a holding isometric mode has more complex neural control strategies [9].

Control strategies of higher complexity could be more vulnerable to disturbing influences. Thus, the AFiso_max_ relative to the maximal strength capacity of a muscle could have the potential to differentiate between a functionally disturbed neuromuscular system and an intact one. An undisturbed, functionally intact neuromuscular system might be able to reach a high AFiso_max_ in relation to the MVIC. As the pre-MVIC and AF_max_ differed insignificantly, AF_max_ could serve as a reference. Cases in which the limb started to give way immediately at the beginning of an AF-measurement (*n* = 3 in the present study), or in which the AFiso_max_ was relatively low (e.g., < 50% of the Max pre-MVIC), the neuromuscular system did not respond adequately to the externally applied force. These inadequate responses could possibly be attributed to inhibitory signals to fusimotor-, skeletomotor- or interneurons within the complex response loops (spinal and supraspinal pathways) [62]. A time delay in the activation of extrafusal muscle fibers is also conceivable. As previously discussed [4], regions involved in the complex motor control are not only the motor cortex itself, but also the thalamus, basal ganglia cerebellum, inferior olivary nucleus, cingulate cortex and the red nucleus. All of these areas process several inputs and can alter motor control. Causes of disturbed control could be highly diverse, including, e.g., nociceptive signals or even emotions [64,65,66,67]. However, the assumptions of neurophysiological influences have to be taken with caution because, in the presented study, an absence of the subject’s attention or bias in the measurement process cannot be ruled out completely (see limitations).

Independently of its cause, a low AFiso_max_ in relation to MVIC or AF_max_ might be a theoretical explanatory approach of the genesis of musculoskeletal complaints and injuries. According to this concept, a low relative AFiso_max_ as an indicator of a disturbed neuromuscular system might be present prior to complaints or injuries. If that holds true in future studies, the detection of AFiso_max_ and its improvement could also play a key role in preventive strategies. Furthermore, an impairment of muscular function is discussed for chronic fatigue syndromes [68], COVID-19 [69], cancer [70] and hormonal dysfunction [71,72]. Currently, e.g., no-load resistance training [73,74] and power training [75] are being investigated, with the aim of improving functional muscle capacity. However, the parameters of Adaptive Force (especially AFiso_max_) might be reduced, too. In this case, improving the AFiso_max_ would be of relevance. We propose that treatments of the possible causes that might impair the AFiso_max_ (see above) might help improve it, rather than training programs alone.

## 5. Conclusions

The presented pneumatic system, as a refinement of the SeBit, is able to measure the MVIC, the MVICpri-con and especially the AF. Conclusions about the MVICpri-con need further examination by use of separate study designs. However, the device is suitable for generating reliable data and can be used to determine the different parameters of Adaptive Force (AFiso_max_, AFecc_max_ and AF_max_). Besides an evaluation of the new device, the study revealed further insights about maximal holding capacity during adaptation to external forces (AFiso_max_). Despite high correlations, it could be discriminated from other maximal forces. Thus, it can be interpreted as a specific and independent parameter of muscle function. The AFiso_max_ should be determined separately and its normalization to the MVIC or AF_max_ might be a meaningful variable in the evaluation of the functionality of the neuromuscular system. Future research can examine whether there is relationship between a low relative AFiso_max_ and the occurrence of injuries or any other complaints. If a relationship is found, the following question arises: How can the AFiso_max_ be improved, especially if the MVIC and/or AF_max_ are interpreted as appropriate, e.g., by a special training program or treatment? It is hypothesized that the holding isometric Adaptive Force is related to complex control processes of the neuromuscular system, and depends on the functional condition of the system itself. This would mean a deficient AFiso_max_ might be treatable by eliminating the causes affecting of this dysfunction.

## Figures and Tables

**Table 1:** Components, measuring equipment and technical specifications of the pneumatic AF system.

Section	Components	Technical Specifications
Basic construction	pivoted and connected levers	range: flexion/extension: 80°–107°
Pressure system	compressor (JUN-AIR 700367; Condor MDR2 EN 60947-4-1)	max. system pressure: 8 bar
	pressure control unit (custom build, Seifert Drucklufttechnik GmbH, Lauter-Bernsbach, Germany)	pressure reduction to max. 2 bar
	bellows cylinder (Zitec SP−2 B04, 2−fach)	Ø 165 mm force: max. 9 kN stroke length: 1–110 m (adjustable) rise time: 0.1–30 s (continuously)
Measuring equipment	1 strain gauge (LMZ 2000N 3006 + amplifier, modified by Biovision, Wehrheim, Germany)	linearly 1 V = 124.74 N
	2 accelerometers + amplifier (modified by Biovision, Wehrheim, Germany)	sensitivity 312 mV/g (range ± 2 g) cosinusoidal between 70°–110° approx. linear linearity: ± 0.2%
	1 pressure sensor (Seifert Drucklufttechnik GmbH, Lauter-Bernsbach, Germany)	linear 1 V = 1.05 bar
	analog to digital converter (National Instruments, modified by Biovision, Wehrheim, Germany)	14-bit range:−5 to 5 V
	software: NI DIAdem	Version 2012
Additional measuring equipment	hydrogoniometer (MT.DOK; Desimed GmbH & Co. KG, Müllheim, Germany)	range: 360° with 2°-intervals

**Table 2:** Group arithmetic means (M) ± standard deviations (SD) of each force type (mean and maximal toques out of 4 measurements) at both days (t_1_ and t_2_) of the total sample of male and female subjects.

	Type of Force	Total Sample M ± SD *n* = 13		Male M ± SD *n* = 9		Female M ± SD *n* = 4	
		t_1_	t_2_	t_1_	t_2_	t_1_	t_2_
mean torques	pre-MVIC	50.70 ± 22.65	50.13 ± 22.50	63.03 ± 14.30	61.89 ± 15.78	22.95 ± 4.89	23.66 ± 3.51
	post-MVIC	46.38 ^b^ ± 21.25	46.91 ^b^ ± 20.76	56.94 ± 16.16	56.80 ± 16.91	22.62 ± 4.80	24.68 ± 2.86
	MVICpri-con	**48.02** ^a^ ± 20.50	**46.58**^a,b^ ± 18.98	58.07 ± 16.09	56.10 ± 14.38	25.41 ± 2.51	25.17 ± 2.58
	AFiso_max_	37.17 ^b^ ± 17.39	35.20 ^b^ ± 17.77	45.61 ± 13.40	42.23 ± 16.53	18.19 ± 6.05	19.36 ± 7.12
	AF_max_	49.49 ± 21.01	48.35 ± 21.66	60.40 ± 14.93	58.79 ± 17.17	24.95 ± 3.35	24.87 ± 5.29
	AFecc_max_	49.39 ± 21.04	48.35 ± 21.66	60.36 ± 14.86	58.79 ± 17.17	24.72 ± 3.15	24.88 ± 5.30
maximal torques	pre-MVIC	**53.67** ^a^ ± 24.83	**51.72**^a^ ± 22.83	66.70 ± 17.13	63.41 ± 16.68	24.34 ± 5.24	25.43 ± 3.61
	post-MVIC	47.47 ^b^ ± 21.89	47.68 ^b^ ± 21.07	58.19 ± 16.97	57.69 ± 17.22	23.34 ± 5.22	25.18 ± 3.11
	MVICpri-con	**49.61** ^a,b^ ± 21.48	**47.92**^a,b^ ± 19.94	59.86 ± 17.47	57.71 ± 15.58	26.56 ± 2.79	25.88 ± 2.86
	AFiso_max_	45.09 ^b^ ± 19.89	43.31 ^b^ ± 20.01	55.76 ± 12.83	52.58 ± 16.35	21.08 ± 5.79	22.47 ± 7.22
	AF_max_	51.20 ± 21.99	50.90 ± 21.93	62.29 ± 16.42	61.53 ± 17.31	26.26 ± 4.03	26.97 ± 4.64
	AFecc_max_	51.10 ± 22.10	50.90 ± 21.93	62.29 ± 16.42	61.53 ± 17.31	25.93 ± 3.73	26.97 ± 4.64

^a^ significant difference (*p* < 0.10) between t_1_ and t_2_ in the total sample (in bold); ^b^ significant difference (*p* < 0.05) to pre-MVIC of the same day of the total sample; males and females differed significantly in all variables (*p* < 0.01); note: due to its similarity to AF_max_, AFecc_max_ was excluded from statistical analyses.

**Table 3:** Inference statistics of mean differences between days (MD t_1_ − t_2_), standard deviations of mean differences (SD_MD_), 90% confidence intervals (90%–CI), *t*-values, degrees of freedom (df), *p*-values, Cohen’s *d_z_* for significant results, standard error of measurements (SEM), minimal important differences (MDC95%), SEM%, SEM95% and intraclass correlation coefficients (ICC(3,1) [95%–CI] of the mean and maximal torques of each force type.

	Type of Force	MD (t_1_ − t_2_) (Nm)	SD_MD_ (Nm)	90%–CI (Nm)	*t*	*df*	*p*	*d_z_*	SEM (Nm)	MDC 95% (Nm)	SEM% (%)	SEM 95% (%)	ICC(3,1) [95%–CI]
mean torques	pre-MVIC	0.57	3.65	−1.23–2.38	0.57	12	0.582	-	2.58	7.15	5.12	10.03	0.987 [0.958–0.996]
	post-MVIC	−0.54	4.19	−2.61–1.53	−0.46	12	0.652	-	2.96	8.20	6.35	12.44	0.980 [0.936–0.994]
	MVICpri-con	1.44	2.59	0.16–2.71	2.01	12	**0.068**	0.56	1.82	5.04	3.67	7.19	0.991 [0.972–0.997]
	AFiso_max_	1.98	8.03	−1.99–5.95	0.89	12	0.392	-	5.68	15.74	15.70	30.77	0.896 [0.694–0.967]
	AF_max_	1.14	2.85	−0.27–2.55	1.44	12	0.175	-	2.01	5.57	4.11	8.05	0.991 [0.971–0.997]
maximal torques	pre-MVIC	1.95	3.94	0.003–3.90	1.79	12	**0.099**	0.50	2.79	7.73	5.29	10.38	0.986 [0.956–0.996]
	post-MVIC	−0.22	4.70	−2.54–2.11	−0.17	12	0.869	-	3.16	8.76	6.64	13.02	0.976 [0.924–0.993]
	MVICpri-con	1.69	2.71	0.35–3.03	2.25	12	**0.044**	0.62	1.92	5.32	3.76	7.36	0.991 [0.972–0.997]
	AFiso_max_	1.77	5.90	−1.15–4.69	1.08	12	0.300	-	4.17	11.56	9.43	18.49	0.956 [0.863–0.986]
	AF_max_	0.31	1.82	−0.50–1.21	0.61	12	0.552	-	1.29	3.58	2.53	4.95	0.996 [0.986–0.999]

Note: A significant difference (*p* < 0.10) between t_1_ and t_2_ is in bold.

**Table 4:** Inference statistics of mean differences (MD) between force types for mean and maximal torques on both days (t_1_ and t_2_), standard deviations of mean differences (SD_MD_), 95% confidence intervals (CI), *t*-values, degrees of freedom (df), *p*-values, Cohen’s *d_z_* for significant results.

Comparison			MD (Nm)	SD_MD_ (Nm)	95% CI (Nm)	*t*	*df*	*p*	*d_z_*
mean torques	t_1_	AFiso_max_−pre-MVIC	−13.52	10.23	−19.70–−7.34	−4.77	12	**<0.001**	1.32
		AF_max_−pre-MVIC	−1.21	4.66	−1.61–4.02	−0.93	12	0.369	-
		AFiso_max_−AF_max_	−12.32	8.10	−17.21–−7.42	−5.48	12	**<0.001**	1.52
		MVICpri-con−pre-MVIC	−2.68	5.47	−0.63–5.98	−1.77	12	0.103	-
		post-MVIC−pre-MVIC	−4.32	4.44	−7.00–1.64	−3.51	12	**0.004**	0.97
	t_2_	AFiso_max_−pre-MVIC	−14.93	11.96	−22.16–−7.70	−4.50	12	**0.001**	1.23
		AF_max_−pre-MVIC	−1.77	5.06	−1.28–4.83	−1.26	12	0.230	-
		AFiso_max_−AF_max_	−13.16	8.83	−18.49–−7.82	−5.37	12	**<0.001**	1.49
		MVICpri-con−pre-MVIC	−3.54	4.37	−6.18–0.91	−2.93	12	**0.013**	0.81
		post-MVIC−pre-MVIC	−3.21	4.01	−5.63–0.79	−2.89	12	**0.014**	0.80
maximal torques	t_1_	AFiso_max_−pre-MVIC	−8.59	8.65	−13.81–−3.36	−3.58	12	**0.004**	0.99
		AF_max_−pre-MVIC	−2.47	5.14	−0.64–5.57	−1.73	12	0.109	-
		AFiso_max_−AF_max_	−6.12	5.75	−9.59–−2.64	−3.84	12	**0.002**	1.06
		MVICpri-con−pre-MVIC	−4.06	6.38	−7.92–0.21	−2.30	12	**0.041**	0.64
		post-MVIC−pre-MVIC	−6.20	4.91	−9.17–3.24	−4.56	12	**0.001**	1.26
	t_2_	AFiso_max_−pre-MVIC	−8.41	9.73	−14.29–−2.53	−3.12	12	**0.009**	0.86
		AF_max_−pre-MVIC	−0.83	4.62	−1.96–3.62	−0.65	12	0.531	-
		AFiso_max_−AF_max_	−7.58	6.28	−11.38–−3.79	−4.36	12	**0.001**	1.21
		MVICpri-con−pre-MVIC	−3.80	4.03	−6.24–1.37	−3.4	12	**0.005**	0.94
		post-MVIC−pre-MVIC	−4.04	3.74	−6.30–1.77	−3.89	12	**0.02**	1.08

Note: A significant difference (*p* < 0.05) is in bold.

## Data Availability

The data presented in this study are available in the main article and supplementary material.

## Supplementary Materials

The following are available online at https://www.mdpi.com/article/10.3390/diagnostics11060923/s1, Figure S1. Scatter plots of the pre- and post-MVIC (maximal voluntary isometric contraction) and MVICpri-con (MVIC with a prior concentric contraction). Figure S2. Scatter plots of AFiso_max_ (maximal isometric Adaptive Force) and AF_max_ (maximal Adaptive Force). Table S1. Torques in Nm of all force types and trials (M1–4) at each day (t_1_ and t_2_).

## Abbreviations

AF	Adaptive Force
AFecc_max_	maximal eccentric AF
AFiso_max_	maximal isometric AF
AF_max_	maximal AF
CV	coefficient of variation
HIMA	holding isometric muscle action
M	mean
Max	maximum
MD	mean difference
MDC95%	minimal detectable change covering 95% of repeated measurements
MVIC	maximal voluntary isometric contraction
pre-MVIC	MVIC at the beginning of the measurement series
post-MVIC	MVIC at the end of the measurement series
MVICpri-con	MVIC with a prior concentric contraction
NI	National Instruments
PIMA	pushing isometric muscle action
SD	standard deviation
SD_MD_	standard deviation of mean differences
SEM	standard error of measurements (random error)
SEM%	random percentage error
SEM95%	random percentage error covering 95% of repeated measurements
t_1_	measuring session at day 1
t_2_	measuring session at day 2

## References

[1] Verkhoshansky Y., Siff M.C. (2009). Supertraining.

[2] Hoff M., Schaefer L., Heinke N., Bittmann F. (2015). Report on adaptive force, a specific neuromuscular function. Eur. J. Transl. Myol..

[3] Schaefer L., Hoff M., Bittmann F. (2017). Measuring system and method of determining the adaptive force. Eur. J. Transl. Myol..

[4] Bittmann F.N., Dech S., Aehle M., Schaefer L.V. (2020). Manual muscle testing—Force Profiles and their reproducibility. Diagnostics.

[5] Schaefer L.V., Bittmann F.N. (2019). Muscular pre-activation can boost the maximal explosive eccentric adaptive force. Front. Physiol..

[6] Rudroff T., Barry B.K., Stone A.L., Barry C.J., Enoka R.M. (2007). Accessory muscle activity contributes to the variation in time to task failure for different arm postures and loads. J. Appl. Physiol..

[7] Rudroff T., Justice J.N., Holmes M.R., Matthews S.D., Enoka R.M. (2010). Muscle activity and time to task failure differ with load compliance and target force for elbow flexor muscles. J. Appl. Physiol..

[8] Garner J.C., Blackburn T., Weimar W., Campbell B. (2008). Comparison of electromyographic activity during eccentrically versus concentrically loaded isometric contractions. J. Electromyogr. Kinesiol..

[9] Schaefer L.V., Bittmann F.N. (2017). Are there two forms of isometric muscle action? Results of the experimental study support a distinction between a holding and a pushing isometric muscle function. BMC Sports Sci. Med. Rehabil..

[10] Schaefer L., Bittmann F. (2020). Mechanotendography in achillodynia shows reduced oscillation variability of pre-loaded Achilles tendon: A pilot study. Eur. J. Transl. Myol..

[11] Bittmann F., Hoff M., Knöchel M., Heinke N., Stolle A. (2011). Adaptive kraft bei epikondylopathie humeri lateralis. Dtsch. Z. Sportmed..

[12] Oranchuk D.J., Nelson A.R., Storey A.G., Diewald S.N., Cronin J.B. (2021). Short-term neuromuscular, morphological, and architectural responses to eccentric quasi-isometric muscle actions. Eur. J. Appl. Physiol..

[13] Arifin W.N. (2018). A web-based sample size calculator for reliability studies. EIMJ.

[14] Bonett D.G. (2002). Sample size requirements for estimating intraclass correlations with desired precision. Stat. Med..

[15] Meldrum D., Cahalane E., Keogan F., Hardiman O. (2003). Maximum voluntary isometric contraction: Investigation of reliability and learning effect. Amyotroph. Lateral Scler. Other Motor Neuron Disord..

[16] World Medical Association Declaration of Helsinki (2001). Ethical principles for medical research involving human subjects. Bull. World Health Organ..

[17] Weir J.P. (2005). Quantifying test-retest reliability using the intraclass correlation coefficient and the SEM. J. Strength Cond. Res..

[18] Cohen J. (1988). Statistical Power Analysis for the Behavioural Sciences.

[19] Atkinson G., Nevill A.M. (1998). Statistical methods for assessing measurement error (reliability) in variables relevant to sports medicine. Sports Med..

[20] Hopkins W.G. (2000). Measures of reliability in sports medicine and science. Sports Med..

[21] Roebroeck M.E., Harlaar J., Lankhorst G.J. (1993). The application of generalizability theory to reliability assessment: An illustration using isometric force measurements. Phys. Ther..

[22] Eliasziw M., Young S.L., Woodbury M.G., Fryday-Field K. (1994). Statistical methodology for the concurrent assessment of interrater and intrarater reliability: Using goniometric measurements as an example. Phys. Ther..

[23] Stratford P.W., Goldsmith C.H. (1997). Use of the standard error as a reliability index of interest: An applied example using elbow flexor strength data. Phys. Ther..

[24] Bartlett J.W., Frost C. (2008). Reliability, repeatability and reproducibility: Analysis of measurement errors in continuous variables. Ultrasound Obstet. Gynecol..

[25] Kropmans T.J.B., Dijkstra P.U., Stegenga B., Stewart R., de Bont L.G.M. (1999). Smallest detectable difference in outcome variables related to painful restriction of the temporomandibular joint. J. Dent. Res..

[26] Nevill A.M., Atkinson G. (1997). Assessing agreement between measurements recorded on a ratio scale in sports medicine and sports science. Br. J. Sports Med..

[27] Bland J., Altman D. (1996). Statistics notes: Measurement error proportional to the mean. BMJ.

[28] Stokes M. (1985). Reliability and repeatability of methods for measuring muscle in physiotherapy. Physiother. Pract..

[29] Ashley C.D., Weiss L.W. (1994). Vertical jump performance and selected physiological characteristics of women. J. Strength Cond. Res..

[30] Brady C., Harrison A., Comyns T. (2018). A review of the reliability of biomechanical variables produced during the isometric mid-thigh pull and isometric squat and the reporting of normative data. Sports Biomech..

[31] Zaki R., Bulgiba A., Nordin N., Ismail N.A. (2013). A systematic review of statistical methods used to test for reliability of medical instruments measuring continuous variables. Iran. J. Basic Med. Sci..

[32] Koo T.K., Li M.Y. (2016). A guideline of selecting and reporting intraclass correlation coefficients for reliability research. J. Chiropr. Med..

[33] Chapman D., Newton M., Nosaka K. (2005). Eccentric torque-velocity relationship of the elbow flexors. Isokinet. Exerc. Sci..

[34] Dartnall T.J., Rogasch N.C., Nordstrom M.A., Semmler J.G. (2009). Eccentric muscle damage has variable effects on motor unit recruitment thresholds and discharge patterns in elbow flexor muscles. J. Neurophysiol..

[35] Prasartwuth O., Taylor J.L., Gandevia S.C. (2005). Maximal force, voluntary activation and muscle soreness after eccentric damage to human elbow flexor muscles. J. Physiol..

[36] Marmon A.R., Pozzi F., Alnahdi A.H., Zeni J.A. (2013). The validity of plantarflexor strength measures obtained through hand-held dynamometry measurements of force. Int. J. Sports Phys. Ther..

[37] Rodrigues F.M., Demeyer H., Hornikx M., Camillo C.A., Calik-Kutukcu E., Burtin C., Janssens W., Troosters T., Osadnik C. (2017). Validity and reliability of strain gauge measurement of volitional quadriceps force in patients with COPD. Chron. Respir. Dis..

[38] Schlumberger A., Schmidtbleicher D. (2000). Grundlagen der kraftdiagnostik in prävention und rehabilitation. Man. Med..

[39] Martin H.J., Yule V., Syddall H.E., Dennison E.M., Cooper C., Sayer A.A. (2006). Is hand-held dynamometry useful for the measurement of quadriceps strength in older people? A comparison with the gold standard biodex dynamometry. GER.

[40] Čeklić U., Šarabon N. (2021). Comparison between Gymnasts and Non-Gymnasts in Isometric Strength of the Lower Limbs. Eur. J. Transl. Myol..

[41] Doss W.S., Karpovich P.V. (1965). A comparison of concentric, eccentric, and isometric strength of elbow flexors. J. Appl. Physiol..

[42] Griffin J.W. (1987). Differences in elbow flexion torque measured concentrically, eccentrically, and isometrically. Phys. Ther..

[43] Seliger V., Dolejš L., Karas V. (1980). A dynamometric comparison of maximum eccentric, concentric, and isometric contractions using EMG and energy expenditure measurements. Eur. J. Appl. Physiol..

[44] Westing S.H., Seger J.Y., Karlson E., Ekblom B. (1988). Eccentric and concentric torque-velocity characteristics of the quadriceps femoris in man. Eur. J. Appl. Physiol..

[45] Westing S.H., Seger J.Y., Thorstensson A. (1990). Effects of electrical stimulation on eccentric and concentric torque-velocity relationships during knee extension in man. Acta Physiol. Scand..

[46] Babault N., Pousson M., Ballay Y., van Hoecke J. (2001). Activation of human quadriceps femoris during isometric, concentric, and eccentric contractions. J. Appl. Physiol..

[47] Amiridis I.G., Martin A., Morlon B., Martin L., Cometti G., Pousson M., van Hoecke J. (1996). Co-activation and tension-regulating phenomena during isokinetic knee extension in sedentary and highly skilled humans. Eur. J. Appl. Physiol..

[48] Singh M., Karpovich P.V. (1966). Isotonic and isometric forces of forearm flexors and extensors. J. Appl. Physiol..

[49] Olson V.L., Smidt G.L., Johnston R.C. (1972). The maximum torque generated by the eccentric, isometric, and concentric contractionsof the hip abductor muscles. Phys. Ther..

[50] Hortobágyi T., Katch F.I. (1990). Eccentric and concentric torque-velocity relationships during arm flexion and extension. Eur. J. Appl. Physiol..

[51] Hunter S.K., Ryan D.L., Ortega J.D., Enoka R.M. (2002). Task differences with the same load torque alter the endurance time of submaximal fatiguing contractions in humans. J. Neurophysiol..

[52] Hortobagyi T., Katch F.I., LaChance P.F. (1989). Interrelationships among various measures of upper body strength assessed by different contraction modes. Eur. J. Appl. Physiol..

[53] Abernethy P.J., Jürimäe J. (1996). Cross-sectional and longitudinal uses of isoinertial, isometric, and isokinetic dynamomety. Med. Sci. Sports Exerc..

[54] Muehlbauer T., Gollhofer A., Granacher U. (2013). Association of balance, strength, and power measures in young adults. J. Strength Cond. Res..

[55] Baker D., Wilson G., Carlyon B. (1994). Generality versus specificity: A comparison of dynamic and isometric measures of strength and speed-strength. Eur. J. Appl. Physiol..

[56] Liu H., Garrett W.E., Moorman C.T., Yu B. (2012). Injury rate, mechanism, and risk factors of hamstring strain injuries in sports: A review of the literature. J. Sport Health Sci..

[57] Van Hooren B., Bosch F. (2017). Is there really an eccentric action of the hamstrings during the swing phase of high-speed running? Part I: A critical review of the literature. J. Sports Sci..

[58] Quatman C.E., Quatman-Yates C.C., Hewett T.E. (2010). A ‘plane’ explanation of anterior cruciate ligament injury mechanisms. Sports Med..

[59] Fong D.T., Chan Y.-Y., Mok K.-M., Yung P.S., Chan K.-M. (2009). Understanding acute ankle ligamentous sprain injury in sports. BMC Sports Sci. Med. Rehabil..

[60] Beeson M.S. (1999). Complications of shoulder dislocation. Am. J. Emerg. Med..

[61] Macefield V.G., Knellwolf T.P. (2018). Functional properties of human muscle spindles. J. Neurophysiol..

[62] Houk J.C., Rymer W.Z. (2011). Neural control of muscle length and tension. Comprehensive Physiology.

[63] Pruszynski J.A., Scott S.H. (2012). Optimal feedback control and the long-latency stretch response. Exp. Brain Res..

[64] Nijs J., Daenen L., Cras P., Struyf F., Roussel N., Oostendorp R.A.B. (2012). Nociception affects motor output: A review on sensory-motor interaction with focus on clinical implications. Clin. J. Pain.

[65] Kugelberg E., Eklund K., Grimby L. (1960). An electromyographic study of the nociceptive reflexes of the lower limb. Mechanism of the plantar responses. Brain.

[66] Sagaspe P., Schwartz S., Vuilleumier P. (2011). Fear and stop: A role for the amygdala in motor inhibition by emotional signals. NeuroImage.

[67] Vogt B.A., Finch D.M., Olson C.R. (1992). Functional heterogeneity in cingulate cortex: The anterior executive and posterior evaluative regions. Cereb. Cortex.

[68] Pietrangelo T., Fulle S., Coscia F., Gigliotti P.V., Fanò-Illic G. (2018). Old muscle in young body: An aphorism describing the Chronic Fatigue Syndrome. Eur. J. Transl. Myol..

[69] Angelini C., Siciliano G. (2020). Neuromuscular diseases and Covid-19: Advices from scientific societies and early observations in Italy. Eur. J. Transl. Myol..

[70] Dalise S., Tropea P., Galli L., Sbrana A., Chisari C. (2020). Muscle function impairment in cancer patients in pre-cachexia stage. Eur. J. Transl. Myol..

[71] Hajjar K., Hagenacker T. (2017). Neuromuscular Disorder as Initial Manifestation of Secondary Hyperparathyroidism—A Case Report. Eur. J. Transl. Myol..

[72] Duyff R.F., Bosch J.V.D., Laman D.M., Van Loon B.-J.P., Linssen W.H.J.P. (2000). Neuromuscular findings in thyroid dysfunction: A prospective clinical and electrodiagnostic study. J. Neurol. Neurosurg. Psychiatry.

[73] Carraro U., Albertin G., Martini A., Giuriati W., Guidolin D., Masiero S., Kern H., Hofer C., Marcante A., Ravara B. (2021). To contrast and reverse skeletal muscle weakness by Full-Body In-Bed Gym in chronic COVID-19 pandemic syndrome. Eur. J. Transl. Myol..

[74] Barbalho M., Coswig V.S., Bottaro M., De Lira C.A.B., Campos M.H., Vieira C.A., Gentil P. (2019). “NO LOAD” resistance training increases functional capacity and muscle size in hospitalized female patients: A pilot study. Eur. J. Transl. Myol..

[75] Šarabon N., Smajla D., Kozinc Ž., Kern H. (2020). Speed-power based training in the elderly and its potential for daily movement function enhancement. Eur. J. Transl. Myol..

