# Supplementary material for: Assessment of the Adaptive Force of Elbow Extensors in Healthy Subjects Quantified by a Novel Pneumatically Driven Measurement System with Considerations of Its Quality Criteria
Source: Diagnostics (Basel). 2021 May 21;11(6):923. doi: 10.3390/diagnostics11060923 (PMC8224031; doi:10.3390/diagnostics11060923)
Supplement: Supplementary file 1 [file diagnostics-11-00923-s001.zip › diagnostics-1202960-supplementary.pdf]

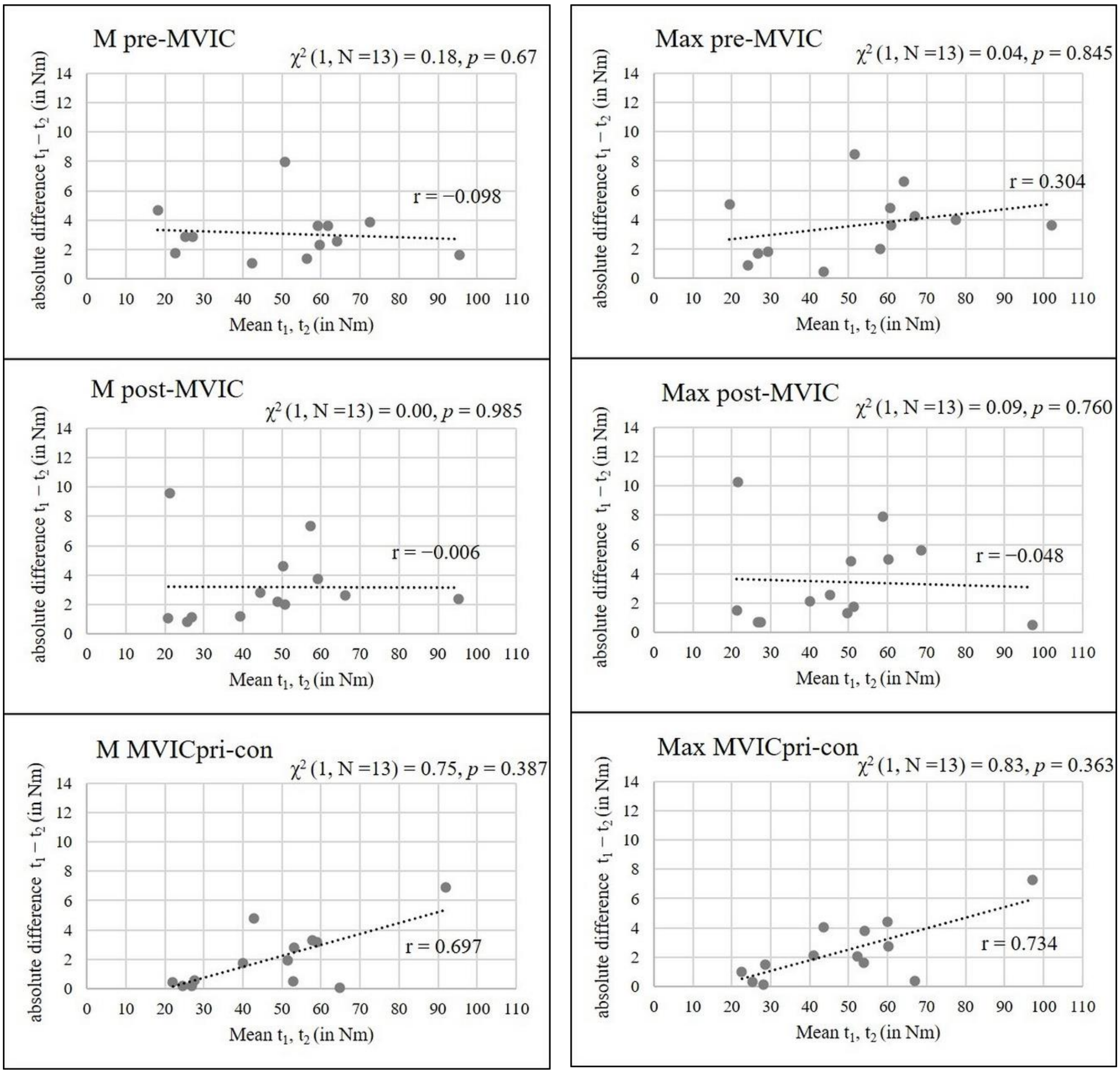

(**a**) (**b**)

Figure S1. Scatter plots of the pre- and post-MVIC (maximal voluntary isometric contraction) and MVICpri-con (MVIC with a prior concentric contraction). The diagrams show the absolute differences between days ($t_1 - t_2$) of (**a**) the mean (M) and (**b**) the maximal (Max) torques out of 4 measurements against the respective measurement means of t_1 and t_2**.** The result of the Breusch-Pagan-test can be found at the top of each panel. Pearson's r is presented besides the regression line. According to Atkinson and Nevill (1998),[1] $r > 0.2$ is rated as a heteroscedastic data.

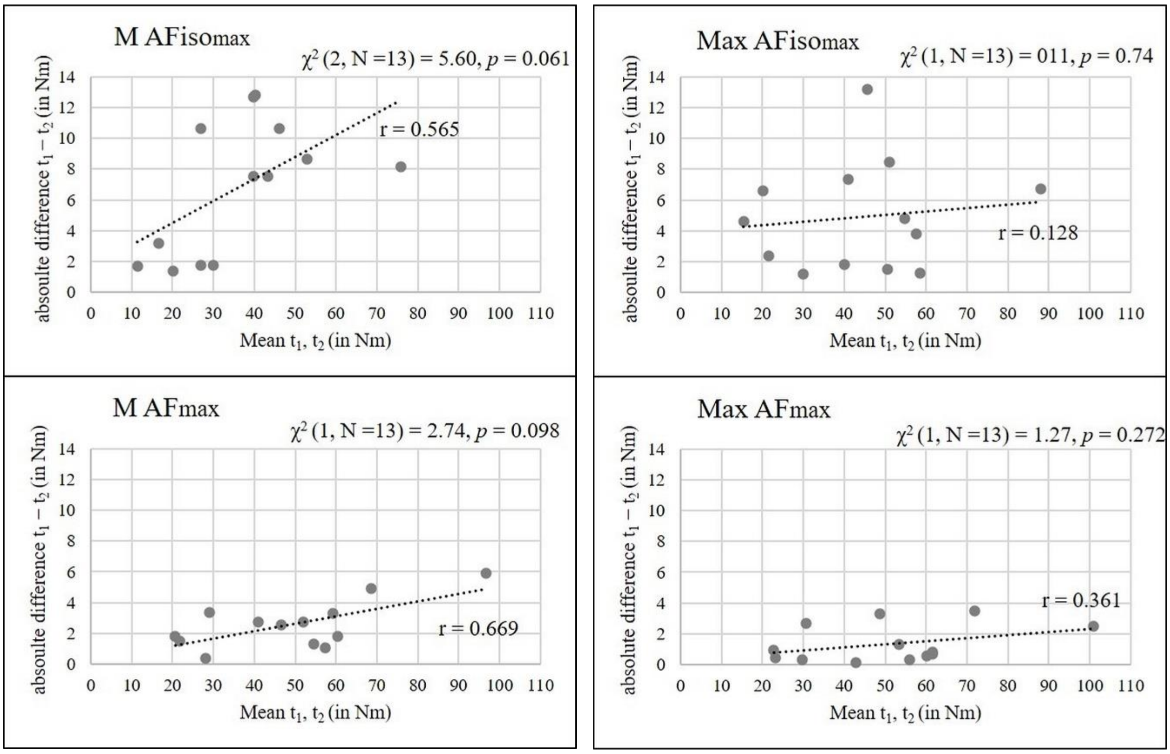

(**a**) (**b**)

Figure S2. Scatter plots of $AFiso_{max}$ (maximal isometric Adaptive Force) and AF_{max} (maximal Adaptive Force). The diagrams show the absolute differences between days ($t_1 – t_2$) of (**a**) the mean (M) and (**b**) the maximal (Max) torques against the respective means out of t_1 and t_2.The result of the White-test (M $AFiso_{max}$) or Breusch-Pagan-test (other variables) can be found at the top of each panel. Pearson's r is presented besides the regression line. According to Atkinson and Nevill (1998),[1] r > 0.2 is rated as a heteroscedastic data.

Table S1. Torques in Nm of all force types and trials (M1 – 4) at each day (t_1 and t_2).

force type	subject (gender)	day 1 (t_1)				day 2 (t_2)			
		**M1**	**M2**	**M3**	**M4**	**M1**	**M2**	**M3**	**M4**
pre-MVIC	1 (f)	22.80	28.44	25.64	25.72	24.70	30.11	30.29	28.96
post-MVIC		27.71	27.10	-	-	26.99	25.54	-	-
MVICpri-con		27.73	28.11	27.79	28.01	27.42	28.26	26.56	27.23
AFiso$_{max}$		13.77	16.80	14.46	14.65	17.71	23.38	10.14	21.28
AFecc$_{max}$		27.25	30.00	27.66	27.84	29.68	27.96	27.61	26.08
AF$_{max}$		27.25	30.00	27.66	27.84	29.68	27.96	27.61	26.08
pre-MVIC	2 (m)	59.77	67.96	69.16	64.51	64.85	64.92	59.06	62.25
post-MVIC		59.34	62.69	-	-	56.86	57.72	-	-
MVICpri-con		61.48	59.96	61.44	59.36	57.31	58.75	57.58	56.04
AFiso$_{max}$		16.04	29.66	57.30	55.20	47.30	52.51	42.00	46.50
AFecc$_{max}$		62.00	61.21	58.81	61.97	55.08	57.29	57.14	61.34
AF$_{max}$		62.00	61.21	58.81	61.97	55.08	57.29	57.14	61.34
pre-MVIC	3 (m)	60.88	63.00	61.86	58.63	57.96	58.18	56.43	57.20
post-MVIC		48.22	47.94	-	-	52.29	53.06	-	-
MVICpri-con		49.64	51.17	50.99	50.01	52.92	51.51	53.22	51.84
AFiso$_{max}$		59.51	58.91	57.17	53.90	55.73	48.42	50.54	40.12
AFecc$_{max}$		59.90	58.44	57.51	55.00	60.49	56.81	54.69	55.19
AF$_{max}$		59.90	58.91	57.51	55.00	60.49	56.81	54.69	55.19
pre-MVIC	4 (f)	23.35	24.56	23.36	23.03	19.40	23.67	22.69	21.40
post-MVIC		22.00	20.90	-	-	20.25	20.52	-	-
MVICpri-con		23.12	21.69	22.02	22.24	21.64	21.85	22.10	21.82
AFiso$_{max}$		20.02	19.48	20.39	18.04	22.78	21.24	20.51	18.83
AFecc$_{max}$		22.19	22.67	23.30	22.12	22.86	21.24	20.72	19.51
AF$_{max}$		22.19	22.67	23.30	22.12	22.86	21.24	20.72	19.51
pre-MVIC	5 (m)	65.91	67.46	58.32	62.62	60.86	60.34	59.69	59.03
post-MVIC		62.67	59.27	-	-	54.76	52.55	-	-
MVICpri-con		60.15	57.56	62.27	58.03	55.61	55.02	56.26	57.84
AFiso$_{max}$		57.74	30.99	59.18	58.22	46.75	57.95	51.73	7.29
AFecc$_{max}$		62.04	60.24	61.71	60.86	59.63	61.24	60.60	56.13
AF$_{max}$		62.04	60.24	61.71	60.86	59.63	61.24	60.60	56.13
pre-MVIC	6 (m)	42.00	43.39	41.22	40.83	43.84	42.94	42.69	42.22
post-MVIC		38.84	41.12	-	-	39.02	38.57	-	-
MVICpri-con		39.37	38.64	38.32	40.02	40.53	40.40	42.16	40.21
AFiso$_{max}$		29.00	39.19	19.43	28.76	41.03	36.60	13.00	32.79
AFecc$_{max}$		42.18	42.79	41.74	42.89	42.79	39.12	38.18	38.48
AF$_{max}$		42.18	42.79	41.74	42.89	42.79	39.12	38.18	38.48
pre-MVIC	7 (m)	82.55	92.93	103.95	98.84	100.31	100.00	86.41	98.10
post-MVIC		90.63	97.36	-	-	96.86	95.89	-	-
MVICpri-con		88.09	95.85	97.17	100.74	89.17	80.94	93.46	90.65
AFiso$_{max}$		84.75	59.79	63.15	79.37	67.05	70.71	91.47	90.29
AFecc$_{max}$		91.33	95.43	87.11	99.67	101.63	102.18	92.56	101.79
AF$_{max}$		91.33	95.43	88.21	99.67	101.63	102.18	92.64	101.79
pre-MVIC	8 (m)	58.99	56.71	59.13	58.96	62.76	61.25	59.49	59.49
post-MVIC		50.47	49.24	-	-	52.23	51.49	-	-
MVICpri-con		53.12	54.69	53.49	50.99	52.73	53.06	52.64	51.89
AFiso$_{max}$		34.75	51.91	47.91	52.18	39.03	30.91	32.93	32.58
AFecc$_{max}$		52.41	53.37	53.67	53.99	52.70	50.43	49.87	49.54
AF$_{max}$		52.41	53.37	53.67	53.99	52.70	50.43	49.87	49.54
pre-MVIC	9 (m)	79.40	67.40	63.61	72.06	74.89	75.41	72.65	75.05
post-MVIC		65.73	63.88	-	-	71.33	63.52	-	-
MVICpri-con		64.66	62.74	66.75	64.73	67.12	62.21	64.49	65.37
AFiso$_{max}$		36.71	-	55.27	46.28	46.84	20.39	46.73	19.78

$AFecc_{max}$		73.13	73.62	69.03	68.34	69.50	70.12	66.30	58.63
AF_{max}		73.13	73.62	69.03	68.34	69.50	70.12	66.30	58.63
pre-MVIC	10 (f)	27.17	26.41	27.50	25.71	23.25	25.82	21.71	24.41
post-MVIC		23.28	27.12	-	-	26.44	25.62	-	-
MVICpri-con		25.35	25.57	25.12	22.33	24.23	25.23	24.45	23.69
$AFiso_{max}$		17.68	17.10	10.32	4.02	13.07	12.00	11.48	5.95
$AFecc_{max}$		21.86	21.34	20.85	22.29	20.70	17.28	17.90	23.25
AF_{max}		21.86	21.34	20.85	22.29	20.70	17.28	17.90	23.25
pre-MVIC	11 (f)	16.89	16.23	16.38	14.00	20.31	20.89	18.98	21.94
post-MVIC		16.35	16.52	-	-	26.76	25.29	-	-
MVICpri-con		25.99	25.77	29.42	26.22	26.23	26.99	27.92	-
$AFiso_{max}$		21.70	25.97	29.45	27.22	30.67	30.36	23.67	26.74
$AFecc_{max}$		24.93	28.14	-	-	-	32.10	30.66	29.65
AF_{max}		24.93	28.14	29.45	27.22	30.67	32.10	30.66	29.65
pre-MVIC	12 (m)	58.15	59.07	58.21	52.90	55.18	57.08	56.53	54.09
post-MVIC		48.96	46.70	-	-	50.30	49.71	-	-
MVICpri-con		54.94	55.98	52.97	54.22	52.21	51.94	52.15	50.53
$AFiso_{max}$		2.42	46.76	49.88	45.43	51.40	39.50	48.79	35.00
$AFecc_{max}$		56.09	55.39	56.15	53.25	55.84	53.20	54.58	51.97
AF_{max}		56.09	55.39	56.15	53.25	55.84	53.20	54.58	51.97
pre-MVIC	13 (m)	55.77	54.64	54.05	54.24	46.60	46.62	46.29	47.29
post-MVIC		45.29	46.50	-	-	42.28	43.92	-	-
MVICpri-con		45.61	45.35	45.05	44.84	40.73	41.58	39.40	40.08
$AFiso_{max}$		40.23	44.55	44.25	0.08	19.33	0.34	29.75	37.21
$AFecc_{max}$		48.00	50.36	47.09	46.13	47.06	42.71	45.28	46.29
AF_{max}		48.00	50.36	47.09	46.13	47.06	42.71	45.28	46.29

AF_{max} = maximal Adaptive Force; $AFecc_{max}$ = maximal eccentric Adaptive Force; $AFiso_{max}$ = maximal isometric Adaptive Force; f = female; m = male; MVIC = maximal voluntary isometric contraction; MVICpri-con = MVIC with a prior concentric contraction.

References

1. Atkinson, G.; Nevill, A.M. Statistical methods for assessing measurement error (reliability) in variables relevant to sports medicine. *Sports Med.* **1998**, *26*, 217–238, doi:10.2165/00007256-199826040-00002.
